# Survey data on household electricity consumption and living status in Northwestern China

**DOI:** 10.1016/j.dib.2016.03.093

**Published:** 2016-04-01

**Authors:** Shuwen Niu, Yanqin Jia, Liqiong Ye, Runqi Dai, Na Li

**Affiliations:** a Key Laboratory of Western China׳s Environmental Systems (Ministry of Education), Lanzhou University, Lanzhou 730000, China; b College of Earth and Environmental Sciences, Lanzhou University, Lanzhou 730000, China; c Lanzhou Business College, Lanzhou 730000, China

**Keywords:** Survey questionnaires, Electricity consumption, Electrical appliances, Data

## Abstract

Based on 1128 survey questionnaires, main information on urban and rural household electricity consumption was obtained. Original data included household income, the price of electricity, all kinds of electrical appliances, purchase price of main appliances, household size, electricity consumption, as well as power, daily use time of electrical appliances in this data article. These data fully reflected behavior, preferences and living pattern of sample households in electricity use and provided the basis for analyzing the relationship between household electricity consumption and the quality of life (“Does electricity consumption improve residential living status in less developed regions? An empirical analysis using the quantile regression approach” [1]).

## Specifications table

**Table:** 

Subject area	*Energy social economics*
More specific subject area	*Electricity consumption, life quality assessment*
Type of data	*Table, figure*
How data was acquired	*Field survey*
Data format	*Raw*
Experimental factors	*N/A*
Experimental features	*Random investigations were carried out at respondents’ homes using a questionnaire*
Data source location	*Northwestern China*
Data accessibility	*Data is within this article*

## Value of the data

• These data contain a lot of useful information on electricity consumption in resident household. This type of information is rare in existing publications.
• The data presented here is useful to analyze the structure and pattern of household electricity consumption.
• The data provides the evidence for understanding the behavior of electricity users.
• The data may be used to conduct comparative analysis with such information in other places.

## 1 Data

The data presented here are table and figure formats. There are 1128 samples. Contents include household per capita income (*PCI*), the price of electricity *(EP)*, the diversity of home appliance (*AD*), purchase price of main appliances (*APC*), household size (*PO*), electricity consumption per capita (*EC*_*p*_) (see data “mmc9.csv” – “mmc13.csv” supplementary files), these data are sorted out from questionnaires. Using these data, we can analyze general statistical characteristics of six variables, correlations among them, and estimate the results of OLS and quantile regressions [2], [3] for 5th, 10th 90th, 95th quantiles in our research article [1].

## 2 Experimental design, materials and methods

The data was obtained by field household survey, we stated work process from questionnaire design, survey location to samples distribution as follows:

### 2.1 Questionnaire design

According to differences in consumption patterns between urban and rural residents [4], two types of questionnaire (urban and rural questionnaires) with different content were designed. Survey contents included home address, number of household member (do not include migrant workers), yearly household income (include salary, service revenue, sale of agricultural products, governmental subsidy, and so on), electricity consumption and electricity price (account on the basis of home electricity receipt), types of and use status of main appliances, willingness to pay for electricity use,. All electrical appliances are listed in questionnaire, investigators add the ticks (√) for the terms that a family owns. Meanwhile, investigators must ask daily time of electrical appliances use.

### 2.2 Data source location

**Fig. 1:**
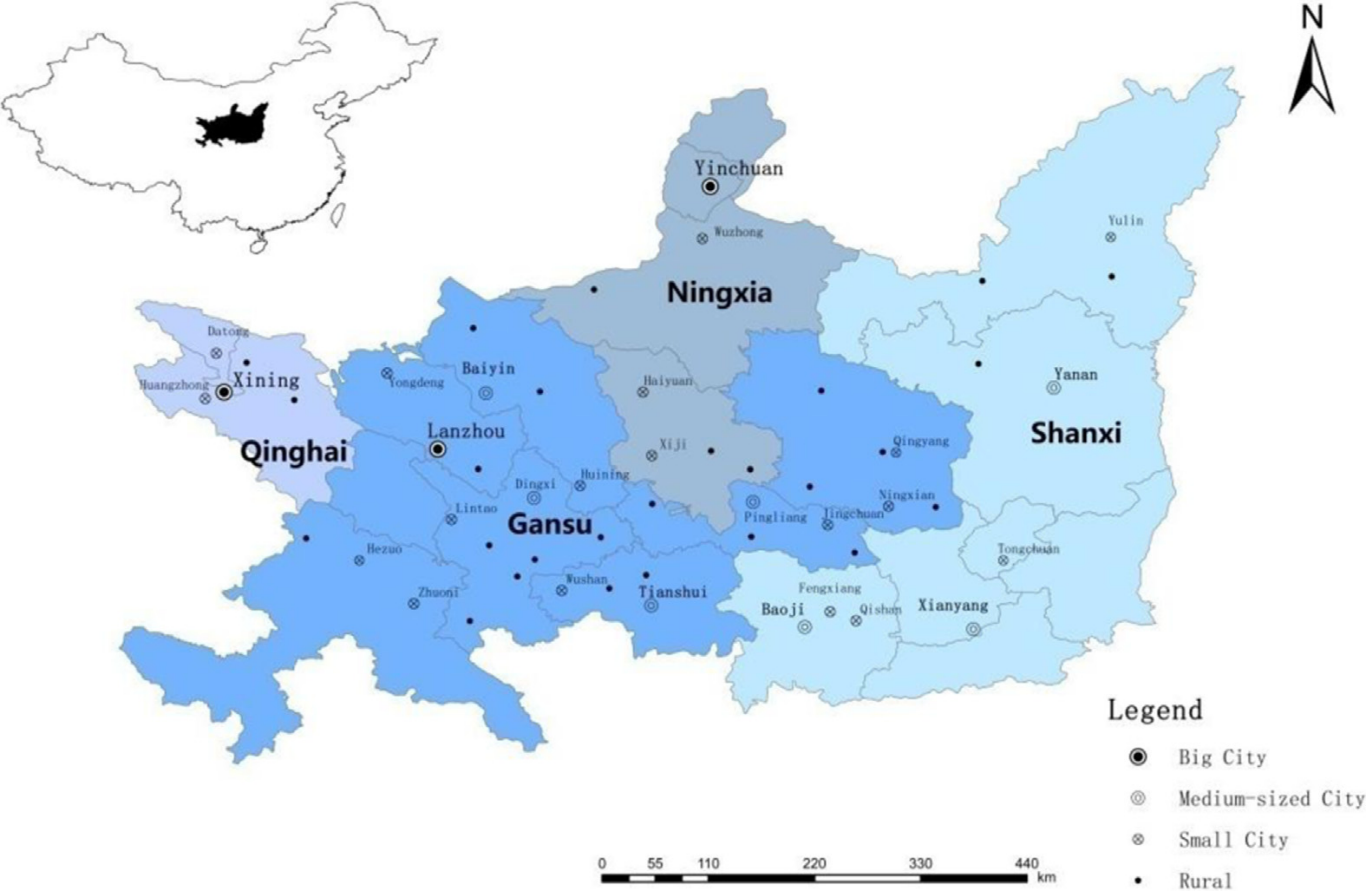
Study area and sample distribution.

Northwestern China has a vast territory. The samples used in this study came from the Eastern Part of Northwestern China. This region is located in the Loess Plateau north of the Qinling Mountains and extends south to the Great Wall [5], east to the Yellow River, and west to Riyue Mountain (at 100°54′–110°29′ degrees East longitude and 33°35′–38°53′ degrees North latitude) (see Fig. 1). This region, with a land area of 255.6 thousand km^2^, is a transition zone between China׳s three natural regions (the eastern monsoon region, the western arid region, and the Qinghai-Tibet Plateau region). The study region contains many different types of climate and terrain. The annual mean temperature is 3.0–13.2 °C. 200–800 mm of rain falls per annum, decreasing from southeast to northwest. The samples were broadly distributed, involving three provincial capitals, 13 prefecture-level cities, and 83 counties in Shanxi Province, Gansu Province, Qinghai Province, and Ningxia Autonomous Region. This region had a population of 37.08 million at the end of 2012, of which the urban population accounted for about 42.02% [6]. In this less developed area of China, the income of urban and rural residents is lower than in the eastern and central regions, and many people still live in poverty [7].

### 2.3 Distribution of survey samples

The survey locations were divided into four types (large cities, medium-sized cities, county towns, and rural areas), and random investigations were carried out at respondents’ homes using a questionnaires. Three large cities, Lanzhou City, Xining City, and Yinchuan City, are the provincial capitals of Gansu, Qinghai, and Ningxia respectively. Five medium-sized cities included Tianshui City and Baiyin City in Gansu Province and Baoji City, Xianyan City, and Yan׳an City in Shanxi Province (see Fig. 1). Survey respondents were chosen in various blocks of each city, with three to five households chosen in the same block. Samples were distributed from the city center to the suburbs. Approximately 100 households were chosen in each large city and about 40 households in each medium-sized city. County towns included 20 small cities or towns, including Tongchuan, Yulin, Fenxiang, Qishan, and Zhidan in Shanxi Province; Anding, Kongntong, Hezue, Wushan, Hueining, Yundeng, Lintao, Zhuoni, Jinchuan, and Ninxian in Gansu Province; Wuzhong, Haiyuan, and Xiji in Ningxia Autonomous Region; and Datong and Huangzhun in Qinghai Province. Approximately 10 households were chosen in each county town. Rural samples were distributed over 40 villages in 26 counties of four provinces (including one autonomous region), with 10–20 households chosen in each village.

### 2.4 The annual household electricity consumption

Combining power and usage time of appliances together, we calculated annual electricity consumption per household (*EC*_*h*_, kW h/a). Table 1 shows electricity consumption of major electrical appliances in five income groups.

## Figures and Tables

**Table 1:** Annual household electricity consumption of major electrical appliances in five income groups.

HEA	Power range (kW)	Low income (133)			Lower-middle income (232)			Middle income (340)			Upper-middle income (234)			High income (189)		
Number	Use time (h a^−1^)	*EC*_*h*_ (kW h)	Number	Use time (h a^−1^)	*EC*_*h*_ (kW h)	Number	Use time (h a^−^^1^)	*EC*_*h*_ (kW h)	Number	Use time (h a^−1^)	*EC*_*h*_ (kW h)	Number	Use time (h a^−1^)	*EC*_*h*_ (kW h)
Decorative lighting	0.04±0.02	213	74	629	252	292	2943	507	432	8767	511	689	14,076	693	824	22,837
Energy-saving lamps	0.02±0.01	424	561	4755	436	567	4945	754	797	12,019	680	438	5951	909	542	9846
Ordinary lamp	0.03±0.01	988	1329	39,380	413	1140	14,126	337	422	4265	324	289	2805	336	118	1190
Fridge	0.12±0.05	121	814	11,917	103	963	12,008	189	986	22,537	184	1212	26,980	196	1274	30,211
Kitchen ventilator	0.12±0.05	28	204	687	74	287	2553	180	298	6443	182	434	9484	196	616	14,490
Rice cooker	0.83±0.03	177	104	15,314	116	91	8837	158	109	14,358	161	176	23,590	196	166	27,145
Electric kettle	1.00±0.5	92	11	998	56	29	1609	162	40	6544	178	61	10,795	129	112	14,395
Water fountain	0.20±0.05	63	16	201	54	172	1856	87	224	3900	98	330	6465	100	538	10,762
Microwave oven	0.70±0.5	14	25	242	39	24	666	70	51	2510	81	73	4125	145	68	6917
Induction cooker	1.35±0.5	108	67	9744	82	83	9225	90	234	28,371	109	258	37,995	151	207	42,225
Refrigerator	1.20±0.5	29	22	783	41	8	370	132	7	1073	136	15	2478	126	38	5729
Soybean milk machine	0.75±0.25	54	0	0	51	20	749	86	37	2376	97	42	3039	148	49	5421
Heated pan	0.80±0.25	35	797	22,316	13	551	5732	68	61	3332	81	0	0	0	0	0
Disinfection cabinet	0.08±0.02	0	0	0	0	0	0	11	204	181	22	285	490	71	358	1984
Electric oven	0.60±0.01	0	0	0	0	0	0	4	4	8	6	7	24	14	20	165
Refrigerated cabinet	0.10±0.02	0	0	0	0	0	0	10	183	183	15	475	712	31	1001	3104
TV	0.12±0.05	412	1304	64,450	175	1503	31,569	209	1144	28,698	204	1279	31,330	211	1058	26,804
Computer	0.27±0.05	55	332	4924	87	324	7617	159	328	14,062	172	464	21,535	192	502	26,035
Video recorder	0.02±0.01	352	285	2207	144	363	1151	186	363	1484	183	159	639	196	96	413
Sound system	0.19±0.02	75	41	584	103	33	649	180	44	1522	179	59	1992	193	50	1828
Air conditioner	0.80±0.5	6	30	145	19	75	1147	42	131	4397	64	207	10,606	81	260	16,856
Electric heater	1.20±0.03	52	38	6973	74	55	4898	140	67	11,337	151	87	15,850	173	102	21,138
Electric fan	0.06±0.01	143	112	1044	106	145	1000	155	190	1918	163	220	2335	182	260	3080
Electric blanket	0.05±0.01	62	60	186	128	51	326	208	30	316	171	0	0	122	0	0
Warm air distributor	1.20±0.03	0	0	0	6	30	213	33	52	2044	45	65	3487	58	86	6008
Washing machine	0.40±0.30	303	46	5538	149	50	2956	189	49	3676	184	68	5011	196	99	7792
Electric shower	1.60±0.4	22	65	2301	45	98	7081	74	125	14,761	96	166	25,455	121	211	40,819
Vacuum cleaner	0.80±0.4			0	0	0	0	0	0	0	23	24	451	33	39	1026
Garment steamer	0.59±0.3		0	0	9	6	32	21	11	141	51	16	472	72	39	1644
Electric iron	1.00±0.2	172	2	385	120	5	656	181	7	1305	179	11	2032	193	13	2532
Electric bicycle	0.35±0.02	37	183	2363	53	201	3724	72	256	6439	81	219	6209	101	110	3871
Electric motorcycle	0.75±0.2	36	110	2957	56	146	6132	78	183	10,676	92	146	10,074	116	73	6351
Total		4053	6971	201,023	2998	7785	134,772	4764	7443	219,645	4883	8395	286,485	5666	9238	362,617
