# Supplementary material for: Survey data on household electricity consumption and living status in Northwestern China
Source: Data Brief. 2016 Apr 1;7:1106–11. doi: 10.1016/j.dib.2016.03.093 (PMC4833132; doi:10.1016/j.dib.2016.03.093)
Supplement: Supplementary file 14 — Supplementary material [file mmc14.docx]

Conflict of Interest Form

We confirm that all data presented in our Data in Brief article is previously unpublished.

There are [no](https://www.baidu.com/s?wd=no&tn=44039180_cpr&fenlei=mv6quAkxTZn0IZRqIHckPjm4nH00T1YYrH99uyw9nWRYuAcvuWcd0ZwV5Hcvrjm3rH6sPfKWUMw85HfYnjn4nH6sgvPsT6KdThsqpZwYTjCEQLGCpyw9Uz4Bmy-bIi4WUvYETgN-TLwGUv3EnHRYnjbsPHbYrjmLrjD1PWn3Ps) conflicts of interest in the data article.

Shuwen Niu, Yanqin Jia, Liqiong Ye, Runqi Dai, Na Li

Lanzhou University, China

2016.1.11
